# Causal effects of circulating cytokine concentrations on risk of Alzheimer’s disease and cognitive function

**DOI:** 10.1016/j.bbi.2022.05.006

**Published:** 2022-08-01

**Authors:** Panagiota Pagoni, Roxanna S. Korologou-Linden, Laura D. Howe, George Davey Smith, Yoav Ben-Shlomo, Evie Stergiakouli, Emma L. Anderson

**Affiliations:** a MRC Integrative Epidemiology Unit, University of Bristol, Bristol, UK; b Population Health Sciences, Bristol Medical School, University of Bristol, Bristol, UK

**Keywords:** Neuroinflammation, Cytokines, Alzheimer’s disease, Cognitive function, Mendelian randomization

## Abstract

**Background:**

There is considerable evidence suggesting a role of neuroinflammation in the pathogenesis of Alzheimer’s disease. Establishing causality is challenging due to bias from reverse causation and residual confounding.

**Methods:**

We used two-sample MR to explore causal effects of circulating cytokine concentrations on Alzheimer’s disease risk and cognitive function. We employed genetic variants from the largest publicly available genome-wide association studies (GWASs) of cytokine concentrations (N = 8,293), Alzheimer’s disease (71,880 cases/383,378 controls), prospective memory (N = 152,605 to 462,302), reaction time (N = 454,157 to 459,523) and fluid intelligence (N = 149,051).

**Results:**

Evidence suggest that 1 standard deviation (SD) increase in levels of CTACK (CCL27) (OR = 1.09 95%CI: 1.01 to 1.19, p = 0.03) increased risk of Alzheimer’s disease. There was weak evidence of a causal effect of MIP-1b (CCL4) (OR = 1.04 95% CI: 0.99 to 1.09, p = 0.08), Eotaxin (OR = 1.08 95% CI: 0.99 to 1.17, p = 0.10), GROa (CXCL1) (OR = 1.04 95% CI: 0.99 to 1.10, p = 0.15), MIG (CXCL9) (OR = 1.17 95% CI: 0.97 to 1.41, p = 0.10), IL-8 (Wald ratio: OR = 1.21 95% CI: 0.97 to 1.51, p = 0.09) and IL-2 (Wald Ratio: OR = 1.21 95% CI: 0.94 to 1.56, p = 0.14) on Alzheimer’s disease risk. A 1 SD increase in concentration of Eotaxin (IVW: OR = 1.05 95% CI: 0.98 to 1.13, p = 0.14), IL-8 (OR = 1.21 95% CI: 1.07 to 1.37, p = 0.003) and MCP1 (OR = 1.07 95% CI: 1.03 to 1.13, p = 0.003) were associated with lower fluid intelligence, and IL-4 (OR = 0.86 95%CI: 0.79 to 0.98, p = 0.02) with higher.

**Conclusions:**

Our findings suggest a causal role of cytokines in the pathogenesis of Alzheimer’s disease and fluid intelligence.

## Highlights

• Evidence suggests a role of neuroinflammation in the pathogenesis of Alzheimer’s disease.
• Mendelian randomization, a causal inference technique, was used to explore this hypothesis.
• Higher levels of several cytokines were associated with increased risk of Alzheimer’s disease.
• Higher levels of several cytokines were associated with fluid intelligence.

## 1 Introduction

Over the last decade attention has been drawn to the interplay between the central nervous system (CNS) and immune responses (i.e. neuroinflammation) (Heneka et al., 2015) in the pathogenesis of Alzheimer’s disease, mostly due to evidence stemming from observational studies suggesting that inflammatory disorders (e.g. rheumatoid arthritis) (Chou et al., 2016) and chronic inflammation (e.g. periodontitis) (Leira et al., 2017) are associated with a higher risk of Alzheimer’s disease. The neuroinflammation hypothesis suggests that in response to production and deposition of Aβ in the brain, the CNS activates microglia to protect the cells and overall brain function. As part of this defense mechanism, secondary inflammatory mediators such as cytokines (Mrak and Griffinbc, 2001, Tuppo and Arias, 2005), lipid metabolites and free radicals are generated to rehabilitate the homeostasis and ensure a healthy neural function (Solito and Sastre, 2012). However, over-activation of microglia may occur and lead to an exaggerated release of pro- and anti-inflammatory mediators (Tejera and Heneka, 2016), resulting in deterioration or even initiation of neurological diseases.

Many observational studies have examined the association between cytokine concentrations and risk of Alzheimer’s disease. A recent *meta*-analysis of 170 studies reported elevated peripheral levels of C-reactive protein (CRP), interleukin-6 (IL-6), interleukin-1 beta (IL-1β), soluble tumour necrosis factor receptor 1 and 2 (sTNFR-1 & sTNFR-2), interleukin-10 (IL-10), monocyte chemoattractant protein-1 (MCP-1) and transforming growth factor-1 (TGF-1) in individuals with an Alzheimer’s disease diagnosis compared to healthy controls (Shen et al., 2019). However, deciphering the role of inflammatory markers in the pathogenesis of Alzheimer’s disease is challenging because of the potential bias due to reverse causation, which refers to the possibility of Alzheimer’s disease being the cause rather than the consequence of inflammation. Confounding is another key source of bias inherent in existing observational studies, as Alzheimer’s disease patients tend to be older, and thus are more at risk of conditions such as obesity and hypertension, which may increase systemic inflammation.

A promising method that could help overcome the limitations of observational studies is Mendelian randomization (MR). Mendelian randomization uses genetic variants as proxies for an exposure and allows investigation of the causal effect of the exposure on the outcome of interest (Davey Smith and Hemani, 2014, Davey Smith and Ebrahim, 2005). Deciphering the causal role of neuroinflammation in the pathogenesis of Alzheimer’s disease could provide valuable information towards possible therapeutic targets. Several studies have used MR to examine this question, but they included only a small, select group of previously implicated cytokines such as TNF-a, interleukins, and CRP, and found limited evidence to support a causal role (Andrews and Goate, 2019, Larsson et al., 2017, Prins et al., 2016, Tsui and Davis, 2018). A recently published MR study explored the causal effect of a wider range of inflammatory markers and suggested there is some evidence of a causal role (Yeung and Schooling, 2021). This study used genetic effects estimated in a GWAS from the UK Biobank in which Alzheimer’s cases were defined based on self-reported family history of dementia, rather than clinical diagnosis and this can reduce power due to heterogeneity. In addition, there have not been any MR studies examining the role of cytokines on cognitive domains relevant to Alzheimer’s disease.

We aimed to examine the causal effects of cytokines on risk of Alzheimer’s disease, prospective memory, reaction time and fluid intelligence using two-sample MR.

## 2 Materials and methods

### 2.1 GWAS summary data

We used the largest publicly available GWAS *meta*-analysis on concentrations of 41 circulating cytokines including up to 8,293 individuals from three independent population cohorts (The Cardiovascular Risk in Young Finns Study (YFS), FINRISK 1997 and FINRISK 2002) (Ahola-Olli et al., 2017). Cytokines GWAS were adjusted for sex, age and body mass index (BMI).

For Alzheimer’s disease the largest publicly available GWAS was used, which consists of three phases. In phase one, 24,087 clinically diagnosed cases from the International Genomics of Alzheimer’s Project (IGAP), the Alzheimer’s Disease Sequencing Project (ADSP) and the Alzheimer’s disease working group of the Psychiatric Genomics Consortium (PGZ-ALZ) and 55,058 matched controls were included. In phase two, cases consist of 47,793 proxy-cases as defined in the UK Biobank and 328,320 proxy-controls (Jansen et al., 2019). Participants were considered proxy-cases if they had positively responded to the question ‘Has your mother or father ever suffered from Alzheimer’s disease/Dementia’. Finally, phase three is a *meta*-analysis of all individuals in phase one & two and therefore consists of 71,880 cases and 383,378 controls of European ancestry. In our main analysis, we used data from phase one because UK Biobank cases included in phase three were not themselves clinically diagnosed with Alzheimer’s disease. As summary data on phase one were not publicly available, we used summary estimates from Korologou- Linden et al. (Korologou-Linden et al., 2020), which corresponds to phase one of the Alzheimer’s GWAS. Effect estimates were adjusted for sex, age, genotyping array and assessment centre.

We used several cognitive function measures in UK Biobank to assess three cognitive domains (i.e., prospective memory, fluid intelligence and reaction time). Briefly, prospective memory was assessed via prospective memory test result (N = 152,605), number of correct matches in round (N = 462,302) and time to correctly identify matches (N = 462,302), reaction time via mean time to correctly identify matches (N = 459,523) and fluid intelligence via fluid intelligence score (N = 149,051). Summary estimates for these cognitive function measures were obtained from IEU GWAS pipeline (Ruth Mitchell, 2019). Additional information about the cognitive function measures can be found in Supplementary Note 1.

### 2.2 Instrument selection

For each cytokine, approximately independent genome-wide significant single nucleotide polymorphisms (SNPs) were identified (r^2^ < 0.01 within a 10,000 kb window, p < 5 × 10^−08^). Eight cytokines had no SNPs available at the genome wide significance threshold, thus we relaxed the significance threshold to p < 5 × 10^−07^ for these. When investigating the causal effects of cytokine concentrations on Alzheimer’s disease and cognitive function measures, we extracted the SD-scaled effect sizes and standard errors for cytokine SNPs from the publicly available cytokine GWAS, and corresponding SD-scaled effect sizes or log odds and standard errors from the outcome GWASs. In cases where genetic variants were not present in the outcome GWAS, we searched for proxy variants using the LDLink online tool (r^2^ < 0.01 within a 10,000 kb window) (Machiela and Chanock, 2015). Before estimating total causal effects, we harmonised the alleles of our datasets and further information about the procedure followed can be found in Supplementary Note 2.

### 2.3 Mendelian randomization analysis

Univariable MR was employed to estimate the total causal effect of each circulating cytokine concentration on Alzheimer’s disease and several cognitive outcomes. MR relies on three assumptions that the genetic variants should satisfy to be considered as valid instruments and therefore yield unbiased causal effect estimates. Genetic variants i) must be strongly associated with the exposure of interest, ii) independently of any confounders of the exposure – outcome association and iii) they are associated with the outcome only via the exposure (i.e. no horizontal pleiotropy) (Lawlor et al., 2008).

When a single variant was available as a proxy for the exposure of interest, the Wald ratio estimator was employed to quantify the causal effect. When multiple variants were available, the Inverse-Variance-Weighted (IVW) method was used to estimate the total causal effect, which is equivalent to fitting a weighted linear regression of the gene-outcome associations on the gene-exposure associations, with the intercept term constrained to zero. Therefore, IVW estimates assume that all genetic variants are valid instruments with no pleiotropic effects (Burgess et al., 2013, Burgess et al., 2015).

### 2.4 Sensitivity analyses

We performed a series of sensitivity analyses to test the validity of the core assumptions which MR relies on. As the validity of MR depends largely on the strength of the genetic instruments, we used the F-statistic to evaluate whether weak instrument bias could have affected our results (Burgess et al., 2011). An F-statistic smaller than 10 indicates that weak instrument bias may be present and causal effect estimates could be biased. Secondly, the estimated total causal effects obtained from IVW method were compared with those obtained from MR-Egger regression and weighted median estimators. Unlike IVW, MR-Egger regression allows for an unconstrained intercept term and provides a robust causal effect estimate, after adjusting for horizontal pleiotropy (Bowden et al., 2015). The weighted median estimator serves as an unbiased causal effect estimate when up to 50% of the instruments are invalid, by estimating the causal effect as the median of the weighted ratio estimates (Bowden et al., 2016). When results from the above methods agree in direction and magnitude, we consider them more likely to be valid. Additionally, the influence of each genetic variant on the outcome was explored by conducting a leave-one out analysis, where genetic variants were systematically removed and causal effects of the remaining SNPs on the outcome were re-estimated (Burgess and Thompson, 2017). In cases where less than three genetic variants were used as instruments, estimation of the MR-Egger and weight median and leave-one out analysis were not feasible. Cochran’s Q statistic was used to assess if the causal estimates of all genetic variants within a single MR analysis were comparable. Substantial heterogeneity is an indication that instruments may not be valid (Bowden et al., 2015). Lastly, to further explore the issue of pleiotropic effects of genetic variants, we repeated analyses using only *cis*-variants (i.e. variants located in the closest proximity to the encoding gene of each cytokine) which are less likely to violate the ‘horizontal pleiotropy assumption’ than variants who are located more distantly (Schmidt et al., 2019). Further information about the procedure followed to extract *cis*-variants and perform *cis*-MR can be found in Supplementary Note 3.

### 2.5 Analyses including proxy cases of Alzheimer’s disease in UKB

The main analysis was conducted using summary data from phase one of the Alzheimer’s disease GWAS, which included only clinically diagnosed cases. To increase the statistical power of our analysis, we re-ran our analysis using summary data from phase three of Alzheimer’s GWAS, which includes proxy cases as defined in UKB (​Jansen et al., 2019).

## 3 Results

### 3.1 Selection of instruments & instrument strength

**Fig. 1:**
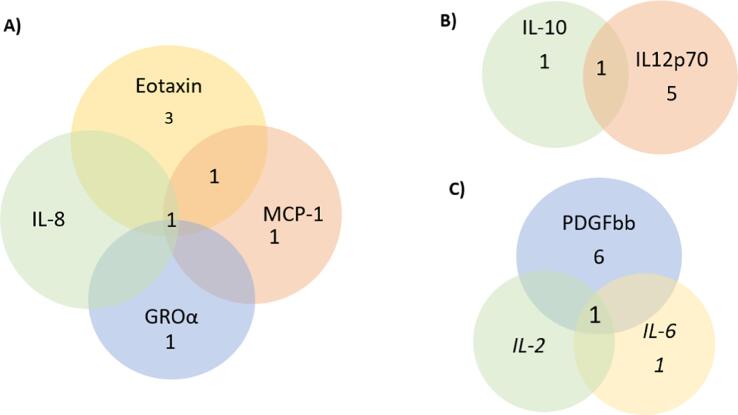
Graphical presentation of the number of genetic variants overlapping between cytokines.

Out of the 41 cytokines we aimed to explore, 26 of them had at least one genetic variant available at the genome-wide significant threshold p < 5 × 10^−08^ for use in our MR analyses. For an additional 8 cytokines, genetic variants were available at a more liberal threshold of p < 5 × 10^−07^. Additional information about the genetic instruments used in our analyses can be found in Table S1. Notably, all selected instruments for cytokine concentrations demonstrated an F-statistic larger than 10, indicating that weak instruments bias is unlikely to bias our results. Information on the number of instruments identified for each cytokine concentration, the threshold used for selecting instruments and the cumulative F-statistic per cytokine can be found in Table 1. Lastly, four genetic variants were identified as genetic instruments for more than one cytokine and Fig. 1 illustrates the overlap. All these variants were included in our main analyses and their influence on our results was further explored in leave – one out analyses.

**Table 1:** Descriptive table of the number of participants for each circulating cytokine concentration, number of instruments identified for each cytokine, the threshold used for selecting instruments and the cumulative F-statistic.

Cytokine abbreviation	Cytokine full name	N**	No. of genetic instruments	Cumulative F-statistic
IL-6*	Interleukin-6	8,189	2	57.4
IL-17	Interleukin-17	7,760	1	38.9
MCP1 (CCL2)	Monocyte chemotactic protein 1	8,293	4	422.5
MIP1b (CCL4)	Macrophage inflammatory protein-1b	8,243	22	2690.8
GROa (CXCL1)	Growth regulated oncogene-alpha	3,505	2	434.8
IFNg*	Interferon gamma	7,701	1	27.6
IL-4*	Interleukin-4	8,124	2	51.5
IL-10	Interleukin-10	7,681	2	336.4
IL-13	Interleukin-13	3,557	2	322.9
IL-7	Interleukin-7	3,409	1	169.8
IL-2ra	Interleukin-2 receptor alpha	3,677	1	167.6
IL12p70	Interleukin-12p70	8,270	4	688.0
IL-16	Interleukin-16	3,483	3	203.6
IL-18	Interleukin-18	3,636	5	299.6
CTACK (CCL27)	Cutaneous T-cell attracting	3,631	4	261.9
Eotaxin	Eotaxin	8,153	5	425.3
HGF	Hepatocyte growth factor	8,292	2	98.0
IP10	Gamma-induced protein 10	3,685	2	63.1
PDGFbb	Platelet derived growth factor BB	8,293	7	549.1
SCF	Stem cell factor	8,290	2	80.4
SCGFb	Stem cell growth factor-beta	3,682	5	321.1
TNFb	Tumour necrosis factor-beta	1,559	2	130.8
TRAIL	TNF-related apoptosis inducing ligand	8,186	12	1914.2
VEGF	Vascular endothelial growth factor	7,118	9	1153.0
MIG (CXCL9)	Monokine induced by interferon- gamma	3,685	1	42.3
RANTES (CCL5)	Regulated on Activation, Normal T Cell Expressed and Secreted	3,421	1	29.9
IL-1ra*	Interleukin −1 receptor alpha	3,638	2	53.40
IL-2*	Interleukin-2	3,475	1	28.2
IL-5	Interleukin-5	3,364	1	37.9
IL-8*	Interleukin-8	3,526	1	25.8
IL-9*	Interleukin-9	3,634	1	25.5
MCP3*	Monocyte specific chemokine 3	843	1	25.6
bNGF	beta nerve growth factor	3,531	1	36.5
MCSF	Macrophage colony-stimulating factor	840	1	31.6

* No genetic variants were available at the p < 5 × 10^−08^ threshold. Thus, genetic variants were identified using a more liberal threshold of p < 5 × 10^−07^.

** Number of participants included in the GWAS of each cytokine concentration.

### 3.2 Causal effects of circulating cytokine concentrations on risk of Alzheimer’s disease and cognitive domains

**Fig. 2A–2E:**
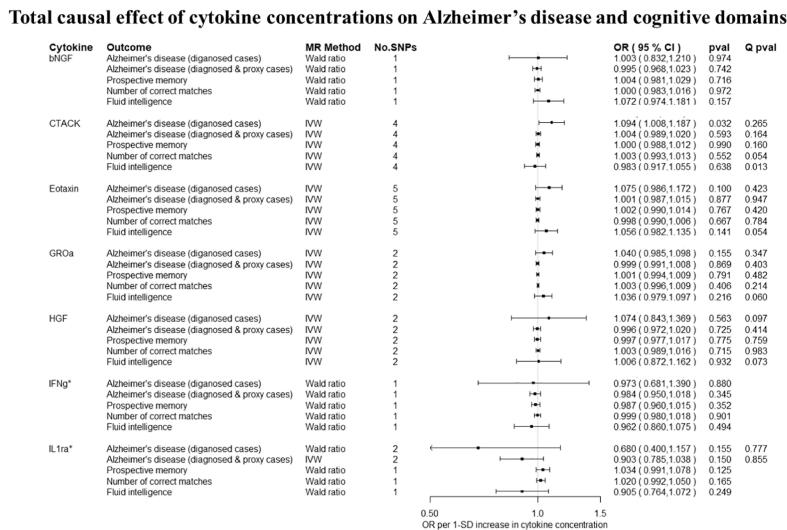

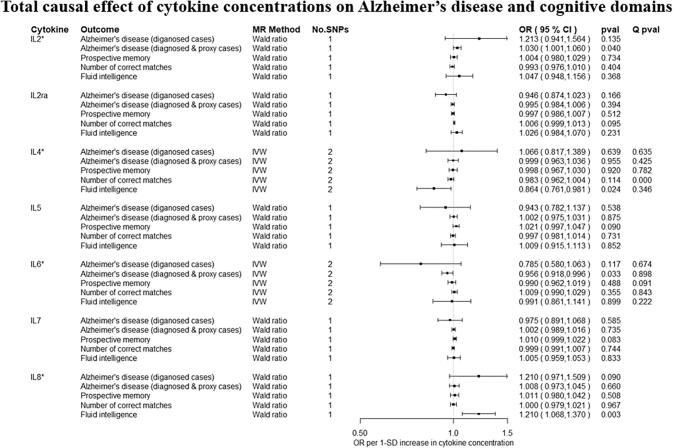

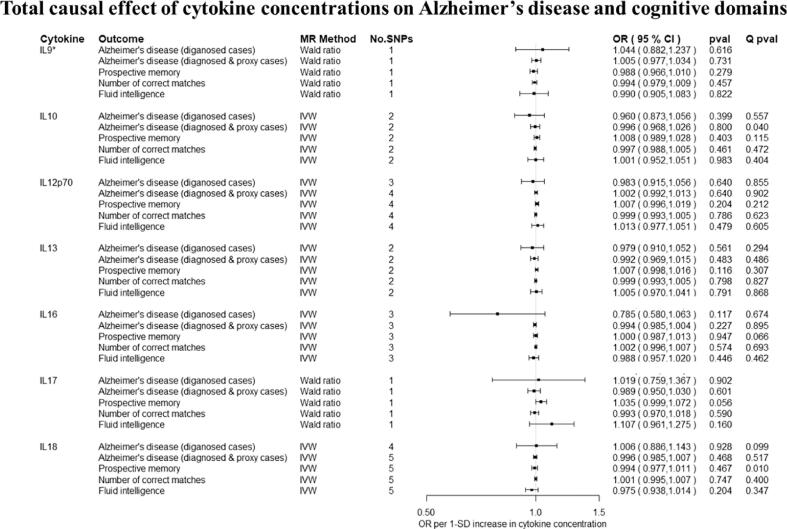

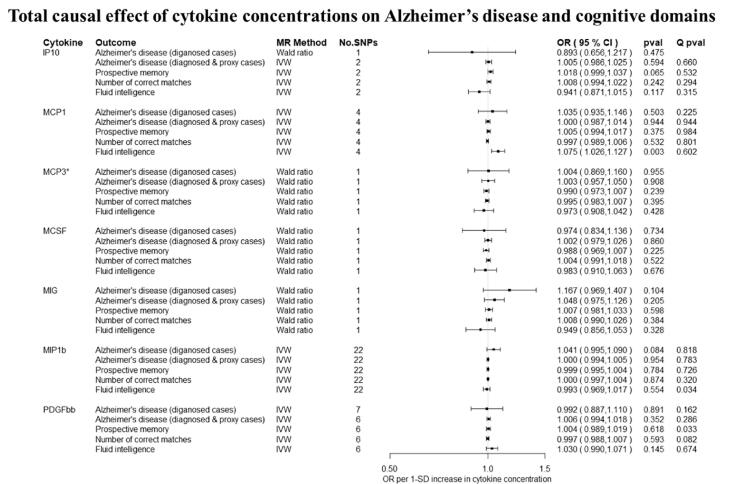

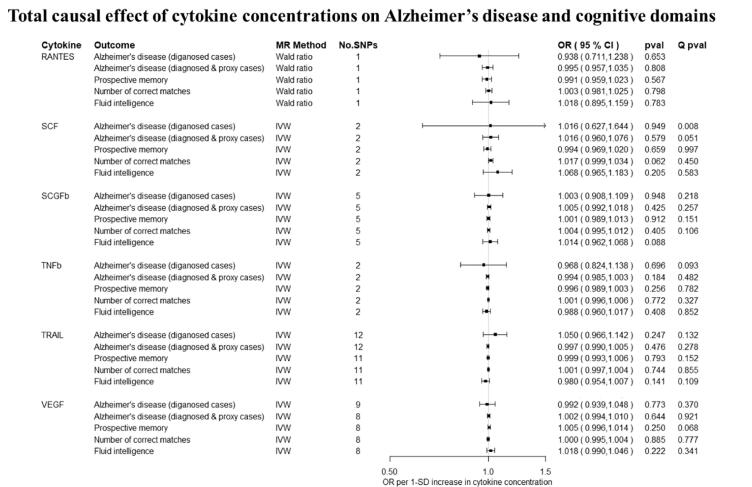
Total causal effect of genetically predicted cytokine concentrations on the risk of Alzheimer’s disease and cognitive outcomes, as estimated by Wald Ratio and IVW. * No genetic variants were available at the p < 5 × 10–08 threshold. Thus, genetic variants were identified using a more liberal threshold of p < 5 × 10–07.

**Fig. 3A–3D:**
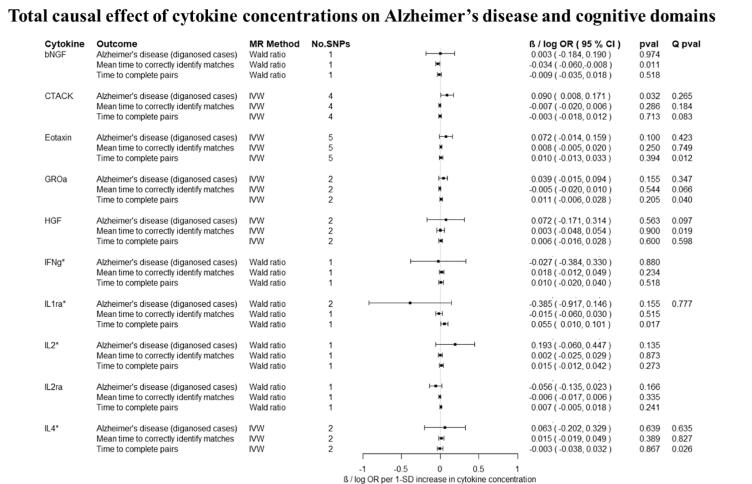

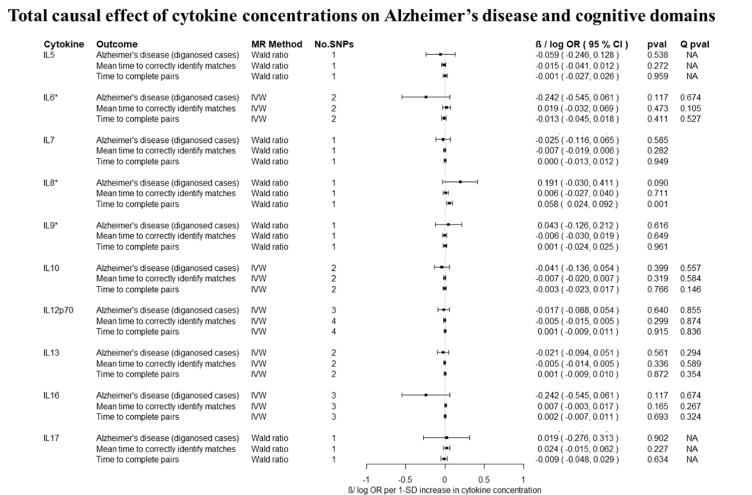

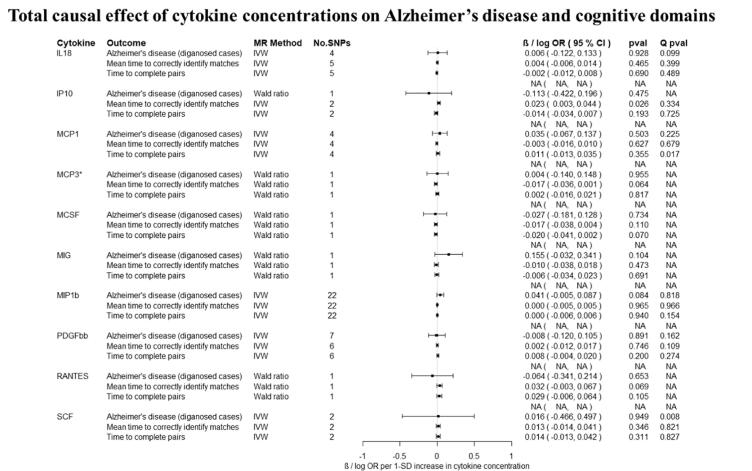

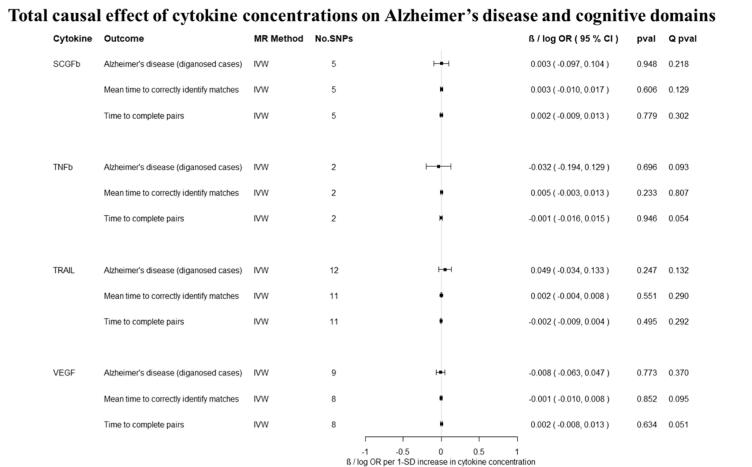
Total causal effect of genetically predicted cytokine concentrations on the risk of Alzheimer’s disease and cognitive outcomes, as estimated by Wald Ratio and IVW. * No genetic variants were available at the p < 5 × 10^−08^ threshold. Thus, genetic variants were identified using a more liberal threshold of p < 5 × 10^−07^.

Overall, there was little evidence to support a causal effect of greater levels of circulating cytokines on risk of Alzheimer’s disease (Fig. 2A–2E, Fig. 3A–3D). Exception was CTACK (CCL27), where we observed evidence of a causal effect on Alzheimer’s disease (IVW: OR per 1 standard deviation (SD) increase = 1.09 95% CI: 1.01 to 1.19, p = 0.03). We also observed weak evidence of a causal effect of 1 SD increase in concentrations of MIP-1b (CCL4) (IVW: OR = 1.04 95% CI: 0.99 to 1.09, p = 0.08) and Eotaxin (IVW: OR = 1.08 95% CI: 0.99 to 1.17, p = 0.10) on risk of Alzheimer’s disease. Additionally, weak evidence was observed for an adverse effect of 1 SD increase in levels of GROa (CXCL1) (IVW: OR = 1.04 95%CI: 0.99 to 1.10, p = 0.15), MIG (CXCL9) (Wald ratio: OR = 1.17 95% CI: 0.97 to 1.41, p = 0.10), IL-8 (Wald Ratio: OR = 1.21 95% CI: 0.97 to 1.51, p = 0.09) and IL-2 (Wald Ratio: OR = 1.21 95% CI: 0.94 to 1.56, p = 0.14).

There was limited evidence to suggest a causal role of greater levels of circulating cytokines on the two of the three cognitive domains (i.e., prospective memory, reaction time) (Fig. 2A–2E, Fig. 3A–3D and 3A–3D). There was weak evidence of a causal effect of greater levels of 4 out of the 34 tested cytokines on fluid intelligence. More specifically, a 1 SD increase in concentration of Eotaxin (IVW: OR = 1.05 95% CI: 0.98 to 1.13, p = 0.14), IL-8 (OR = 1.21 95% CI: 1.07 to 1.37, p = 0.003) and MCP1 (OR = 1.07 95% CI: 1.03 to 1.13, p = 0.003) were causally linked with lower fluid intelligence score, while IL-4 (OR = 0.86 95% CI: 0.79 to 0.98, p = 0.02) with a higher fluid intelligence score.

### 3.3 Sensitivity analyses

When more than three genetic instruments were available and MR-Egger and Weighted median could be estimated, we observed comparable results to the IVW. More specifically, for the cytokines we observed evidence of a causal effect on Alzheimer’s disease risk, the MR-Egger and Weighted median estimators yielded similar effect estimates to the IVW estimator (CTACK: MR-Egger slope: OR = 0.98 95% CI: 0.83 to 1.17, p = 0.88; Weighted median: OR = 1.10 95% CI: 1.01 to 1.20, p = 0.03; MIP-1b: MR-Egger slope: OR = 1.01 95% CI: 0.93 to 1.09, p = 0.79; Weighted median: OR = 1.01 95% CI: 0.94 to 1.07, p = 0.83; Eotaxin: MR-Egger slope: OR = 1.06 95% CI: 0.86 to 1.31, p = 0.58; Weighted median: OR = 1.11 95% CI: 1.00 to 1.24, p = 0.05) (Supplementary material Fig. S1A – F). There was limited evidence of heterogeneity as estimated by Cochran’s Q statistic and after iteratively removing genetic variants results remained virtually the same in the leave-one-out analysis.

When we restricted our analyses to variants located in closest proximity to the encoding gene of each cytokine, we obtained at least one *cis* variant for 11 cytokines that were initially included in our main analysis. Overall, results remained virtually the same, but with wider confidence intervals, as smaller numbers of instruments were included (Supplementary material Fig. S2A–B). For greater levels of the TRAIL cytokine, we observed weak evidence of a detrimental effect (IVW: OR = 1.23 95% CI: 0.97 to 1.55, p = 0.07) on Alzheimer’s disease risk.

### 3.4 Analyses including proxy cases of Alzheimer’s disease in UKB

Overall, there was limited evidence to suggest a causal effect of circulating cytokine concentrations on risk of Alzheimer’s disease (Fig. 2A–2E, Fig. 3A–3D, Fig. 1, Fig. 2A–2E, Fig. 3A–3D). As the sample size increased significantly with the addition of proxy-cases, point estimates of causal effects were comparable, but confidence intervals were narrower compared to analyses including only cases of Alzheimer’s disease. For IL-2, a detrimental causal effect on Alzheimer’s disease risk was observed (Wald Ratio: OR = 1.03 95% CI: 1.00 to 1.06, p = 0.04), although the effect estimate was much smaller in magnitude compared to analyses including only diagnosed cased of Alzheimer’s disease.

## 4 Discussion

We investigated the effect of 34 circulating cytokine concentrations on Alzheimer’s disease risk and three cognitive domains (i.e., prospective memory, fluid intelligence and reaction time). We observed some evidence for a detrimental effect of greater levels of CTACK (CCL27), MIP-1b (CCL4), Eotaxin, GROa (CXCL1), MIG (CXCL9), IL-8 and IL-2 on the risk of Alzheimer’s disease. Additionally, Eotaxin, MCP1 and IL-8 were associated with lower fluid intelligence, while IL-4 was associated with higher fluid intelligence.

Previous two-sample MR studies have investigated the causal role of a few cytokines on risk of Alzheimer’s disease and reported limited evidence to support a causal role (Andrews and Goate, 2019, Larsson et al., 2017, Prins et al., 2016, Tsui and Davis, 2018). However, those studies were underpowered and larger Alzheimer’s disease GWAS have since been published (as used in our study). Moreover, no previous MR studies have examined the causal effects of circulating cytokine concentrations on cognitive domains.

Chemokines are cytokines which regulate immune cell migration and are thought to be mediators of the peripheral monocytes into the inflamed CNS (Zuena et al., 2019), and are thus hypothesized to be involved in the pathogenesis of Alzheimer’s disease (Guedes et al., 2018). CTACK (CCL4), MIG (CXCL9), GROa (CXCL1), MIP-1b (CCL4), Eotaxin (CCL11) and IL-8 belong to the chemokine family and evidence suggest that they could potentially play a role in Alzheimer’s disease. More specifically, CTACK (CCL27) is a chemokine involved in the CNS as it is expressed in the cerebral cortex and limbic regions which are mainly affected in Alzheimer’s disease (Gunsolly et al., 2010). Previous studies have observed higher levels of circulating CTACK (CCL27) in Alzheimer’s disease patients compared to healthy controls (Gongora-Rivera et al., 2020, Lee et al., 2008). However, future research is required to better characterise the role of CTACK (CCL27) in Alzheimer’s disease aetiology.

MIG (CXCL9) is another chemokine that is considered to play a role in the interplay between neurons and glial cells, and binds onto the CXCR3 receptor which has been previously reported to be involved in the pathogenesis of various CNS conditions (e.g. multiple sclerosis, glioma, bipolar disorder) (Koper et al., 2018, Zhou et al., 2019). Regarding Alzheimer’s disease, a cross-sectional study reported substantially higher levels of circulating MIG (CXCL9) in patients with Alzheimer’s disease compared to non-cognitively impaired and mildly-cognitively impaired participants (Lee et al., 2008). Additionally, a recent case-control study demonstrated evidence for an association between higher levels of MIG and Alzheimer’s disease in a Mexican population (Gongora-Rivera et al., 2020).

A case-control study found that GROa (CXCL1) is overexpressed in the brains of 23 Alzheimer’s disease patients, with no prior diagnosis of immunological diseases, hypertension, cardiac disease or diabetes, compared to age-matched controls (Zhang et al., 2013). This result is supported by animal studies, where CXCL1 was found to drive the hypermethylation of Tau in the primary cortical neurons of mice (Xia and Hyman, 2002, Shang et al., 2019). This is supporting to our findings and together, it suggests a plausible causal role of CXCL1 in the pathogenesis of Alzheimer’s disease.

Xia et al. reported that receptors of MIP-1b (CCL4) were present on microglia and subpopulation of reactive astrocytes and neurons in brains of patients with Alzheimer’s disease compared to controls, thus they could potentially play a role in the progression of Alzheimer’s disease through glial-glial and glial-neuronal interactions (Xia et al., 1998). Moreover, higher levels of MIP-1b have been associated with cognitive decline in patients with Alzheimer’s disease (Taipa et al., 2019). Additionally, several studies have reported that expression of CCL4 is greater in brains of HIV-infected patients with dementia compared to HIV-infected patients without dementia, which indicates that CCL4 possibly regulates an inflammatory process that indirectly affects neurons (Minagar et al., 2002, Schmidtmayerova et al., 1996, Sui et al., 2005).

Higher levels of Eotaxin were identified in the cerebrospinal fluid and serum of Alzheimer’s disease cases compared to healthy controls (Taipa et al., 2019, Choi et al., 2008, Soares et al., 2012). In contrast, in two cohort studies, higher levels of Eotaxin in the plasma were not associated with Alzheimer’s disease progression (Westin et al., 2012, Leung et al., 2013). However, all the studies are of small sample size and thus underpowered to identify associations.

Evidence regarding the role of IL-8 in the Alzheimer’s disease pathogenesis is contradicting. A case-control study observed elevated levels of IL-8 in the CSF of Alzheimer’s disease patients compared to non-demented controls (Taipa et al., 2019), when another case-control study reported significantly lower levels in both serum and CSF of Alzheimer’s disease patients (Hesse et al., 2016). Additionally, IL-8 plasma and CSF levels have been found to be associated with higher levels of p-tau and with higher levels of CSF Aβ1-42, which are hallmarks of Alzheimer’s disease (Bettcher et al., 2018). However, evidence stems from studies with limited number of participants and further research is required to elucidate the role of these chemokines in the pathogenesis and progression of Alzheimer’s disease.

### 4.1 Strengths & limitations

The main strength of the present study is the use of two-sample MR which could aid in overcoming the drawbacks of traditional observational epidemiology (i.e., reverse causation, residual confounding), thus allowing the estimation of the effect size of the association between circulating cytokines and Alzheimer’s disease. Additionally, the plethora of cytokines which we could identify instruments for allowed us to explore causal associations between cytokine concentrations and Alzheimer’s disease. Following an agnostic approach could be beneficial in this setting as limited observational evidence exists for most of the studied cytokines. We also used the largest publicly GWAS study of Alzheimer’s disease, which included 24,087 clinically diagnosed cases and thus, our study had statistical power to identify causal effects. The power to detect an odds ratio of 1.1 per 1 – SD increase in circulating cytokine concentration, with a = 0.05 and a coefficient of determination (R^2^) of 3% to 6%, was between 60% and 80%.

There are a few limitations of this study. As few of the genetic variants used in our MR analyses were used as instruments for more than one cytokine, we cannot exclude the possibility that our results were biased due to horizontal pleiotropic effects. We addressed this issue by estimating the total causal effects with alternative MR methods (MR- Egger and weighted median). Even though results remained virtually the same we could not completely exclude the possibility of pleiotropic effects as these methods are not reliable when a limited number of genetic variants is available, which is the case for many of the cytokines investigated.

Moreover, cytokines are complex in their activity as they act pleiotropically (i.e. single cytokine acts on several different cell types), are redundant in their activity (i.e. same process might be activated by multiple cytokines) and can act either synergistically or antagonistically (Zhang and An, 2007). This complex activity of cytokines raises two main issues in our study. The first issue is the possibility that the observed causal effects are either confounded or mediated by one or more cytokines, and therefore not representing the direct causal effect of each cytokine (i.e., the causal effect after taking into account the causal effect of all other cytokines that might mediate or confound the observed total causal effect). A future direction in deciphering the direct causal effects of cytokines on risk of Alzheimer’s disease would be the employment of multivariable Mendelian randomization, which allows the estimation of direct causal effects (Burgess et al., 2015, Sanderson et al., 2019). However, we were not able to implement MVMR mostly due to the presence of correlation between the estimated genetic effects of the inflammatory markers, as they were obtained from the same participants. Additionally, the limited number of genetic instruments available for each cytokine would have resulted in weak instrument bias and would have limited the statistical power to identify direct effects in the MVMR framework (Sanderson et al., 2020). The second issue is that in our analyses we assume no gene-gene or gene-environment interactions and thus modelling the interplay between the multiple inflammatory markers examined is not feasible.

Another limitation relates to the measures used to assess cognitive function in UKB. In contrast to the gold standard assessment, which is typically carried out by a trained psychologist, in UKB a fully automated touchscreen assessment was used. The reliability and validity of such methods have been previously questioned. However, it has been found that UKB tests and well-validated tests for assessment of the same cognitive domain were moderately to strongly correlated, therefore UKB tests demonstrate adequate validity (Fawns-Ritchie et al., 2020, Lyall et al., 2016). Additionally, the test reliability was adequate across time for most of the UKB tests, except the pairs matching test (i.e. number of correct matches in round) and prospective memory test, where test–retest correlations were lower compared to well-validated tests (54).Lastly, the observed causal effects might be the consequence of collider bias. Collider bias occurs when conditioning on a variable that is affected by both exposure and outcome and could lead to spurious associations (Gkatzionis and Burgess, 2019, Paternoster et al., 2017). In our study, the GWAS used to extract instruments for cytokine concentrations was adjusted for BMI, which is affected by both cytokines and Alzheimer’s disease, thus it could be considered as a collider. However, this is unlikely to affect our results as individuals in the cytokines GWAS were not selected based on their BMI measurement.

## 5 Conclusion

In a two-sample MR framework, we observed some evidence to support a causal role of cytokines in the pathogenesis of Alzheimer’s disease. More studies are needed to elucidate the specific mechanistic pathways that underlie this process. A better understanding of these processes could potentially lead to novel therapeutic targets for affected individuals.

## Data Availability Statement

Summary GWAS data for circulating cytokine concentrations were available at [https://www.ebi.ac.uk/gwas/publications/27989323]. Summary GWAS data for Alzheimer’s disease (Phase 3) were accessed from [https://ctg.cncr.nl/software/summary_statistics]. Summary GWAS data for prospective memory, fluid intelligence and reaction time were obtained from IEU GWAS pipeline [https://data.bris.ac.uk/data/dataset/pnoat8cxo0u52p6ynfaekeigi].

## Funding

This work was supported by a grant from the BRACE Alzheimer’s Disease charity (BR16/028). PP, ELA, and ES work in a unit that receives funding from the University of Bristol and the UK Medical Research Council (MC_UU_00011/1, MC_UU_00011/3, MC_UU_00011/6). RKL was supported by a Wellcome Trust PhD studentship (Grant ref: 215193/Z18/Z). ELA is funded by an MRC Skills Development Award from the UK Medical Research Council (MR/P014437/1). LDH is funded by a Career Development Award from the UK Medical Research Council (MR/M020894/1). This publication is the work of the authors, and ELA, will serve as a guarantor for the contents of this paper.

## Declaration of Competing Interest

The authors declare that they have no known competing financial interests or personal relationships that could have appeared to influence the work reported in this paper.
