# Supplementary material for: Causal effects of circulating cytokine concentrations on risk of Alzheimer’s disease and cognitive function
Source: Brain Behav Immun. 2022 Aug 1;104:54–64. doi: 10.1016/j.bbi.2022.05.006 (PMC10391322; doi:10.1016/j.bbi.2022.05.006)
Supplement: Supplementary data 1 [file mmc1.docx]

**SUPPLEMENTARY MATERIAL**

**SUPPLEMENTARY NOTES**

**Supplementary Note 1.** Description of cognitive function measures in UK Biobank.

**Supplementary Note 2.** Harmonization of alleles.

**Supplementary Note 3.** *Cis*-Mendelian randomization.

**SUPPLEMENTARY TABLES**

**Table S1.** Characteristics of genetic variants associated with each circulating cytokine concentration.

**Table S2.** Location of each encoding gene and the number of resulting cis variants included in our *cis* – Mendelian randomization.

**SUPPLEMENTARY FIGURES**

**Figure S1A-F.** Total causal effects of circulating cytokine concentrations on the risk of Alzheimer’s disease and several cognitive outcomes, as estimated by Wald Ratio, IVW, MR-Egger and Weighted median estimators.

**Figure S2A-B.** Total causal effects of circulating cytokine concentrations on the risk of Alzheimer’s disease and several cognitive outcomes, as estimated by Wald Ratio and IVW estimators in the *cis* - Mendelian randomization analysis.

**SUPPLEMENTARY NOTES**

**Supplementary Note 1.** Description of cognitive function measures in UK Biobank.

| **Cognitive domain** | **Cognitive function measure** | **Description** | **Field ID** |
| --- | --- | --- | --- |
| Prospective memory | Prospective memory test result | Participants were asked to take a set of cognitive tests. Before the tests started, participants were instructed that at the end of the tests four coloured shapes will appear at the screen and despite they will be asked to touch the Blue square they should touch the Orange circle instead.  This variable was analysed as an ordinal categorical variable, with lowest category reflecting better prospective memory test result and highest category reflecting worse prospective memory test result. | 20018 |
| Prospective memory | Number of correct matches in round | Participants were shown a set of picture cards and asked to remember as many of them as possible. Then the cards were turned over and the participants had to identify correct matching pairs.  This variable was analysed as an ordinal variable, with lowest category reflecting higher number of correct matches and highest category reflecting lower number of correct matches. | 398 |
| Prospective memory | Time to complete round | This is the time participants took to correctly identify matching pairs.  This variable was analysed as continuous variable. | 400 |
| Reaction time | Mean time to correctly identify matches | Participants were shown two cards at a time and instructed to press the button on the box as quickly as possible when the symbols on the cards match. This exercise involved 12 pairs of cards.  This variable was analysed as continuous variable. | 20023 |
| Fluid intelligence | Fluid intelligence score | Participants were instructed to answer as many intelligence questions as possible (maximum 13) within 2 minutes.  This variable was analysed as an ordinal variable, with lowest category reflecting higher fluid intelligence score and highest category reflecting lower fluid intelligence score. | 20016 |

**Supplementary Note 2.** Harmonization of alleles.

Before estimating total causal effects, we harmonised our datasets so that the effect estimates of each exposure were coded to express the effect estimate per increasing allele, so that outcome alleles correspond to the same alleles as in the exposure. For genetic variants which are ambiguous (i.e., the forward strand is not clear) the effect allele frequencies of both exposure and outcome are required to employ the harmonization. However, in our analysis allele frequencies were not available in the GWAS of cytokines concentrations and therefore alignment of alleles between exposure and outcome was not feasible in a few cases, resulting in the exclusion of those ambiguous genetic variants due to being “palindromic”.

**Supplementary Note 3.** *Cis*-Mendelian randomization.

We defined as *cis* variants all genetic variants located within a 500kb window at either side of the gene that encodes each cytokine. When *cis* variants were available for a cytokine, we further selected independent genome-wide significant variants (r^2^<0.01 within a 10,000 kb window, p<5×10^-08^) and corresponding SD-scaled effect sizes and standard errors were extracted from the publicly available datasets [1]. After finalizing the list of *cis* variants for each cytokine, we extracted the corresponding log odds or effect estimates and standards errors from the Alzheimer’s disease and cognitive outcomes GWAS. Lastly, we performed harmonisation of alleles and estimated the total causal effects of each cytokine concentration on the risk of Alzheimer’s disease and cognitive outcomes using the same statistical analysis as in our main analysis (i.e., Wald ratio, IVW, MR-Egger, Weighted Median). More information about the location of each encoding gene and the number of resulting *cis* variants can be found in Table S3.

**SUPPLEMENTARY TABLES**

| **Table S1.** Characteristics of genetic variants associated with each circulating cytokine concentration. | | | | | | |
| --- | --- | --- | --- | --- | --- | --- |
| **Cytokine abbreviation** | **SNP ID** | **Chromosome** | **trans/cis** | **Effect/ Ref allele** | **Beta (SE)** | **P-value** |
| IL-6* | **rs13412535** | 2 | trans | G/A | 0.1164 (0.0215) | 7.34E-08 |
|  | rs72831623 | 17 | trans | A/G | 0.1973 (0.0372) | 1.08E-07 |
| IL-17 | rs1530455 | 3 | trans | T/C | 0.1080 (0.0173) | 4.87E-10 |
| MCP-1 (CCL2) | **rs12075** | 1 | trans | A/G | 0.2185 (0.0155) | 1.44E-44 |
|  | rs2036297 | 3 | trans | A/G | 0.1190 (0.0160) | 1.09E-13 |
|  | **rs2228467** | 3 | trans | C/T | 0.2637 (0.0291) | 9.19E-20 |
|  | rs7632755 | 3 | trans | A/G | 0.2938 (0.0316) | 1.18E-20 |
| MIP1b (CCL4) | rs113010081 | 3 | trans | C/T | 0.5954 (0.0236) | 3.85E-140 |
|  | rs2673050 | 3 | trans | T/G | 0.1314 (0.0161) | 3.14E-16 |
|  | rs4683315 | 3 | trans | G/A | 0.1347 (0.0236) | 8.97E-09 |
|  | rs73074316 | 3 | trans | G/A | 0.1436 (0.0216) | 2.80E-11 |
|  | rs79091774 | 3 | trans | C/A | 0.4606 (0.0751) | 8.83E-10 |
|  | rs10491120 | 17 | trans | A/G | 0.3001 (0.0318) | 5.15E-21 |
|  | rs111942332 | 17 | trans | G/T | 0.4727 (0.0573) | 1.70E-16 |
|  | rs113877493 | 17 | cis | C/T | 0.6124 (0.0218) | 1.62E-173 |
|  | rs117453826 | 17 | trans | G/A | 0.5774 (0.0593) | 5.07E-22 |
|  | rs117620244 | 17 | trans | C/T | 0.3528 (0.0495) | 1.87E-12 |
|  | rs117715247 | 17 | cis | G/A | 0.3510 (0.0590) | 3.09E-09 |
|  | rs17693183 | 17 | trans | G/A | 0.5795 (0.0795) | 8.93E-13 |
|  | rs2190980 | 17 | trans | G/A | 0.1067 (0.0167) | 1.43E-10 |
|  | rs4795162 | 17 | Trans | A/G | 0.1261 (0.0158) | 1.14E-15 |
|  | rs4796072 | 17 | trans | G/T | 0.1149 (0.0177) | 8.12E-11 |
|  | rs60516659 | 17 | cis | A/G | 0.2691 (0.0248) | 3.64E-27 |
|  | rs6505501 | 17 | trans | C/T | 0.1556 (0.0191) | 3.71E-16 |
|  | rs7221878 | 17 | trans | C/T | 0.3045 (0.0463) | 7.37E-11 |
|  | rs76842834 | 17 | trans | C/T | 0.4206 (0.0472) | 7.33E-19 |
|  | rs76960253 | 17 | cis | T/C | 0.5233 (0.0585) | 5.45E-19 |
|  | rs80007108 | 17 | trans | A/C | 0.2259 (0.0310) | 2.73E-13 |
|  | rs9330240 | 17 | trans | C/T | 0.4745 (0.0460) | 5.84E-25 |
| GROa (CXCL1) | **rs12075** | 1 | trans | A/G | 0.3751 (0.0237) | 1.24E-55 |
|  | rs508977 | 4 | cis | G/T | 0.3802 (0.0280) | 7.56E-42 |
| IFNg* | rs78296352 | 1 | trans | T/G | 0.3430 (0.0652) | 1.38E-07 |
| IL-4* | rs17713451 | 7 | trans | A/G | 0.1274 (0.0253) | 4.97E-07 |
|  | rs10512267 | 9 | trans | C/T | 0.0824 (0.0161) | 2.94E-07 |
| IL-10 | rs282258 | 2 | trans | T/C | 0.0992 (0.0162) | 1.00E-09 |
|  | **rs4349809** | 6 | trans | T/G | 0.2853 (0.0165) | 5.77E-67 |
| IL-13 | rs75438658 | 6 | trans | C/T | 0.3430 (0.0625) | 4.12E-08 |
|  | rs9472168 | 6 | trans | A/G | 0.4244 (0.0248) | 1.08E-65 |
| IL-7 | rs4320361 | 6 | trans | G/T | 0.3245 (0.0249) | 6.87E-39 |
| IL-2ra | rs12722497 | 10 | cis | A/C | 0.6279 (0.0485) | 1.57E-38 |
| IL12p70 | rs12199215 | 6 | trans | T/C | 0.1278 (0.0192) | 5.11E-11 |
|  | rs145023524† | 6 | trans | A/G | 0.2790 (0.0394) | 1.54E-12 |
|  | **rs4349809** | 6 | trans | T/G | 0.3777 (0.0159) | 2.56E-124 |
|  | rs7754905 | 6 | trans | G/A | 0.1029 (0.0190) | 4.28E-08 |
| IL-16 | rs4253283 | 4 | trans | T/C | 0.1460 (0.0262) | 1.75E-08 |
|  | rs1801020 | 5 | trans | A/G | 0.1733 (0.0272) | 4.53E-10 |
|  | rs4778636 | 15 | cis | G/A | 0.7272 (0.0633) | 1.11E-30 |
| IL-18 | rs385076 | 2 | trans | C/T | 0.2432 (0.0248) | 1.66E-22 |
|  | rs115267715† | 2 | trans | C/T | 0.4508 (0.0080) | 1.72E-08 |
|  | rs116656892 | 5 | trans | T/C | 0.5298 (0.0925) | 1.05E-08 |
|  | rs17229943 | 5 | trans | C/A | 0.3120 (0.0463) | 1.62E-11 |
|  | rs71478720 | 11 | cis | C/T | 0.2669 (0.0276) | 3.71E-22 |
| CTACK (CCL27) | rs2070074 | 9 | cis | A/G | 0.4467 (0.0374) | 1.79E-32 |
|  | rs58704839† | 9 | cis | A/G | 0.1785 (0.0284) | 3.29E-10 |
|  | rs55764737 | 15 | trans | T/C | 0.5313 (0.0972) | 4.62E-08 |
|  | rs135564 | 22 | trans | G/A | 0.1893 (0.0268) | 2.43E-12 |
| Eotaxin | **rs12075** | 1 | trans | A/G | 0.1671 (0.0156) | 1.33E-26 |
|  | **rs2228467** | 3 | trans | C/T | 0.4163 (0.0292) | 2.27E-46 |
|  | rs3091309 | 3 | trans | A/G | 0.1283 (0.0203) | 3.63E-10 |
|  | rs342511 | 3 | trans | A/G | 0.0927 (0.0157) | 3.60E-09 |
|  | rs2024050 | 7 | cis | A/G | 0.1728 (0.0303) | 1.10E-08 |
| HGF | rs3748034 | 4 | trans | T/G | 0.1495 (0.0234) | 1.81E-10 |
|  | rs5745687 | 7 | cis | C/T | 0.3072 (0.0406) | 2.75E-14 |
| IP10 | rs113831257 | 4 | cis | A/G | 0.3592 (0.0644) | 2.53E-08 |
|  | rs9450351† | 6 | trans | T/C | 0.2768 (0.0489) | 1.48E-08 |
| PDGFbb | rs12990266† | 2 | trans | A/G | 0.2363 (0.0342) | 3.18E-12 |
|  | rs13024765 | 2 | trans | C/T | 0.1014 (0.0158) | 1.14E-10 |
|  | **rs13412535** | 2 | trans | A/G | 0.3352 (0.0214) | 2.46E-55 |
|  | rs2324229 | 6 | trans | T/C | 0.0894 (0.0161) | 3.48E-08 |
|  | rs28406863 | 15 | trans | G/T | 0.2089 (0.0382) | 4.78E-08 |
|  | rs4965869 | 15 | trans | T/C | 0.1840 (0.0181) | 5.66E-24 |
|  | rs9806745 | 15 | trans | A/C | 0.1162 (0.0163) | 1.10E-12 |
| SCF | rs1557570 | 1 | trans | T/G | 0.1186 (0.0170) | 2.74E-12 |
|  | rs4841899 | 9 | trans | C/T | 0.1004 (0.0178) | 1.78E-08 |
| SCGFb | rs4656185 | 1 | trans | A/G | 0.2050 (0.0256) | 1.16E-15 |
|  | rs17876031 | 5 | trans | G/A | 0.1514 (0.0255) | 2.25E-09 |
|  | rs117716477 | 12 | trans | A/C | 0.8384 (0.0841) | 1.34E-23 |
|  | rs73185877 | 12 | trans | A/G | 0.5249 (0.0711) | 1.18E-13 |
|  | rs116924815 | 19 | trans | T/C | 0.6079 (0.0738) | 1.74E-16 |
| TNF-b | rs116196280 | 1 | trans | T/G | 0.7179 (0.1006) | 4.98E-13 |
|  | rs78296352 | 1 | trans | T/G | 1.2215 (0.1366) | 4.76E-21 |
| TRAIL | rs3136596 | 3 | cis | A/G | 0.1147 (0.0209) | 3.65E-08 |
|  | rs79287178 | 3 | cis | G/A | 0.4317 (0.0421) | 9.12E-25 |
|  | rs11081739† | 18 | trans | A/G | 0.1411 (0.0202) | 3.34E-12 |
|  | rs141603697† | 18 | trans | T/C | 0.7497 (0.0752) | 4.50E-23 |
|  | rs193112415 | 18 | trans | C/T | 1.0421 (0.0623) | 2.15E-62 |
|  | rs57396456 | 18 | trans | C/T | 0.5626 (0.0518) | 1.25E-27 |
|  | rs62093514 | 18 | trans | T/C | 1.0618 (0.0552) | 6.86E-82 |
|  | rs62093947 | 18 | trans | C/T | 0.7596 (0.046) | 3.31E-61 |
|  | rs74778900 | 18 | trans | T/C | 0.5906 (0.0532) | 2.59E-28 |
|  | rs77451439 | 18 | trans | G/A | 0.4322 (0.0369) | 1.21E-31 |
|  | rs9952273 | 18 | trans | T/C | 0.8640 (0.0499) | 3.86E-69 |
| VEGF | rs41282660 | 6 | cis | G/A | 0.1613 (0.0263) | 1.33E-09 |
|  | rs4507572 | 6 | cis | T/C | 0.1007 (0.0171) | 3.34E-09 |
|  | rs67798973 | 6 | cis | A/G | 0.1389 (0.0175) | 1.29E-15 |
|  | rs6920532 | 6 | cis | T/C | 0.1803 (0.0267) | 8.68E-12 |
|  | rs6921438 | 6 | cis | G/A | 0.4900 (0.0175) | 2.09E-171 |
|  | rs74675876 | 6 | cis | C/T | 0.2822 (0.0366) | 7.62E-15 |
|  | rs9381249† | 6 | cis | C/T | 0.2482 (0.0397) | 3.09E-10 |
|  | rs9472183 | 6 | cis | G/A | 0.1282 (0.017) | 5.19E-14 |
|  | rs34881325 | 9 | trans | C/T | 0.1082 (0.0189) | 1.04E-08 |
| MIG (CXCL9) | rs55876513† | 4 | cis | T/G | 0.1660 (0.0255) | 8.23E-11 |
| RANTES (CCL5) | rs74472919 | 13 | trans | T/C | 0.3313 (0.0605) | 3.97E-08 |
| IL-1ra* | rs146151667 | 2 | trans | A/G | 0.5072 (0.0978) | 3.36E-07 |
|  | rs35803309† | 7 | trans | A/AT | 0.2080 (0.0404) | 2.36E-07 |
| IL-2* | **rs13412535** | 2 | trans | A/G | 0.1764 (0.0332) | 1.18E-07 |
| IL-5 | rs7767396 | 6 | trans | A/G | 0.1515 (0.0246) | 7.69E-10 |
| IL-8* | rs12075 | 1 | trans | A/G | 0.1200 (0.0236) | 3.88E-07 |
| IL-9* | rs76963786 | 12 | trans | C/T | 0.2865 (0.0557) | 4.50E-07 |
| MCP3* | rs10892381 | 11 | trans | T/C | 0.2412 (0.0476) | 3.56E-07 |
| bNGF | rs28637706 | 19 | trans | G/T | 0.1589(0.0263) | 1.42E-09 |
| MCSF | rs56367447 | 8 | trans | C/T | 0.4967(0.0883) | 1.72E-08 |
| * No genetic variants were available on p< 5×10^-08^ threshold. Thus, genetic variants were identified using a more liberal threshold of p< 5×10^-07^. | | | | | | |
| † rs11081739, rs9381249, rs35803309, rs12990266 were not available in cognitive outcomes GWAS and no proxies were found  rs145023524, rs9450351 were not available in Alzheimer’s disease GWAS (Phase 1) and no proxies were found  rs115267715 was not available in Alzheimer’s disease GWAS (Phase 1 & Phase 3) and no proxy was found  rs141603697 was used as a proxy for rs58704839 in the Alzheimer’s disease analysis (Phase1 & Phase3)  rs1857821 was used as a proxy for rs55876513 in the cognitive outcomes analysis  rs10972195 was used as a proxy for rs58704839 in the cognitive outcomes analysis | | | | | | |

| **Table S2.** Location of each encoding gene and the number of resulting cis variants included in our *cis* – Mendelian randomization | | |
| --- | --- | --- |
| **Cytokine abbreviation** | **Gene location** | **No. SNPs** |
| IL2ra | Chr 10: 6,052,652 - 6,104,333 | 1 |
| VEGF | VEGFA: Chr6: 43,737,921-43,754,224  VEGFB: Chr11: 64,002,010-64,006,259  VEGFC: Chr4: 177,604,689-177,713,895 | -  8  - |
| IL-16 | Chr 15: 81,451,916-81,606,399 | 1 |
| IL-18 | Chr 11: 112,013,974-112,034,840 | 1 |
| GROa (CXCL1) | Chr 4: 74,735,110-74,737,025 | 1 |
| MIP1b (CCL4) | Chr17: 34,430,983-34,433,014 | 6 |
| TRAIL | Chr 3: 172,223,298-172,241,265 | 2 |
| HGF | Chr 7: 81,328,326-81,399,754 | 1 |
| Eotaxin | Chr 7: 75,440,983-75,452,674 | 1 |
| CTACK (CCL27) | Chr 9: 34,661,877-34,664,045 | 2 |
| MIG (CXCL9) | Chr 4: 76,922,428-76,928,662 | 1 |

**SUPPLEMENTARY FIGURES**

| 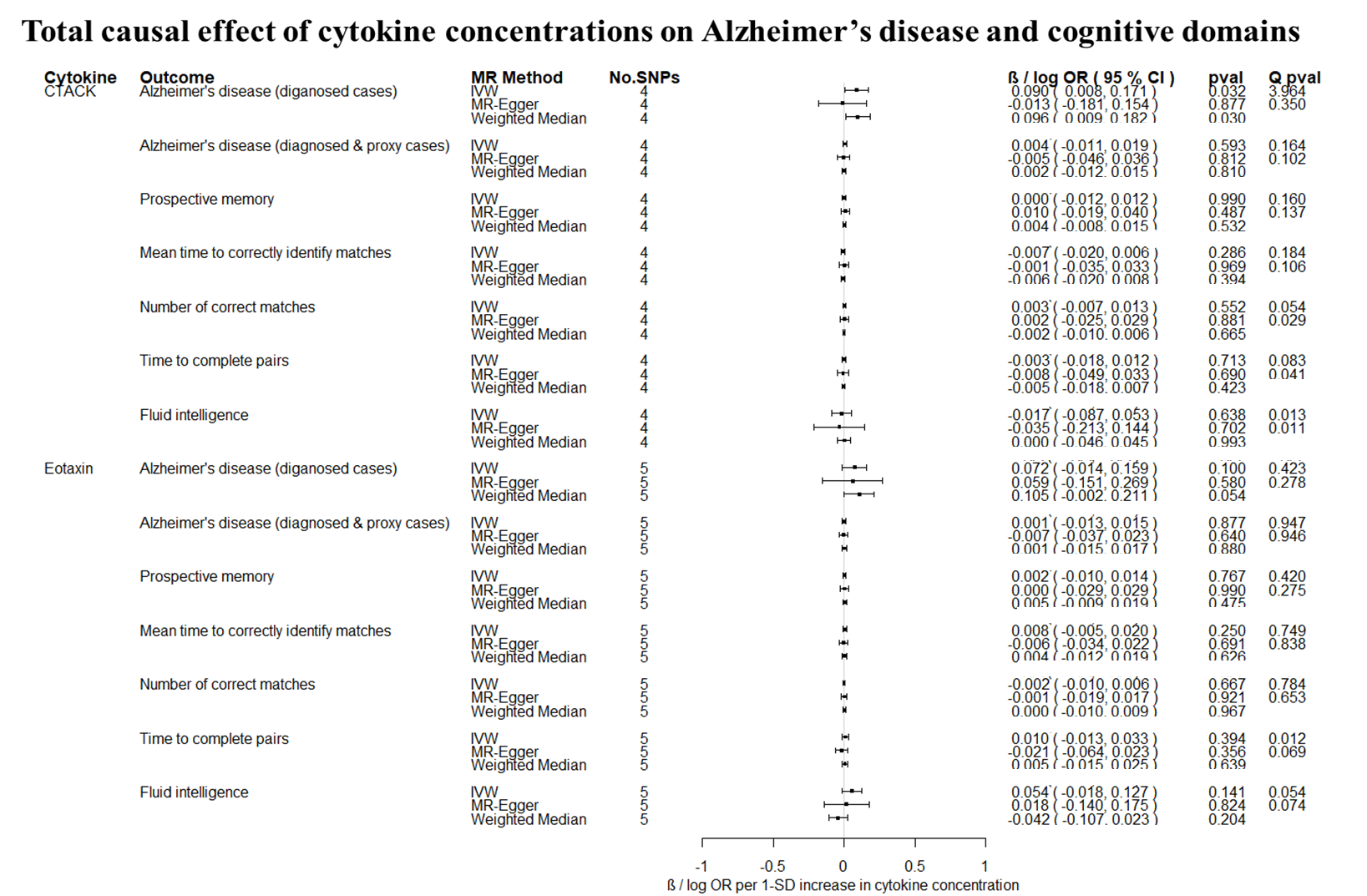 |
| --- |
| **Figure S1A.** Total causal effects of circulating cytokine concentrations on the risk of Alzheimer’s disease and several cognitive outcomes, as estimated by Wald Ratio, IVW, MR-Egger and Weighted median estimators. |

| 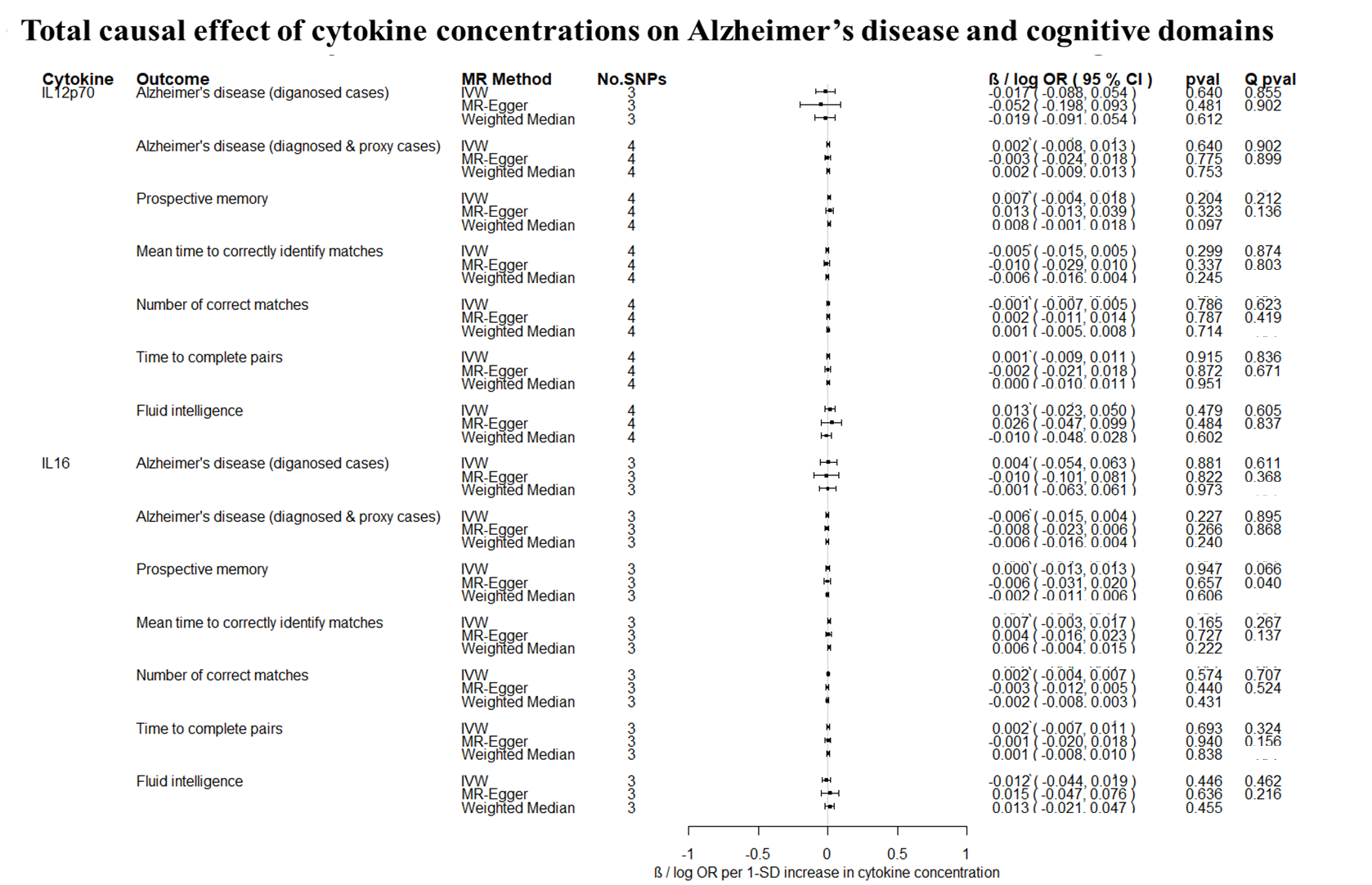 |
| --- |
| **Figure S1B.** Total causal effects of circulating cytokine concentrations on the risk of Alzheimer’s disease and several cognitive outcomes, as estimated by Wald Ratio, IVW, MR-Egger and Weighted median estimators. |

| 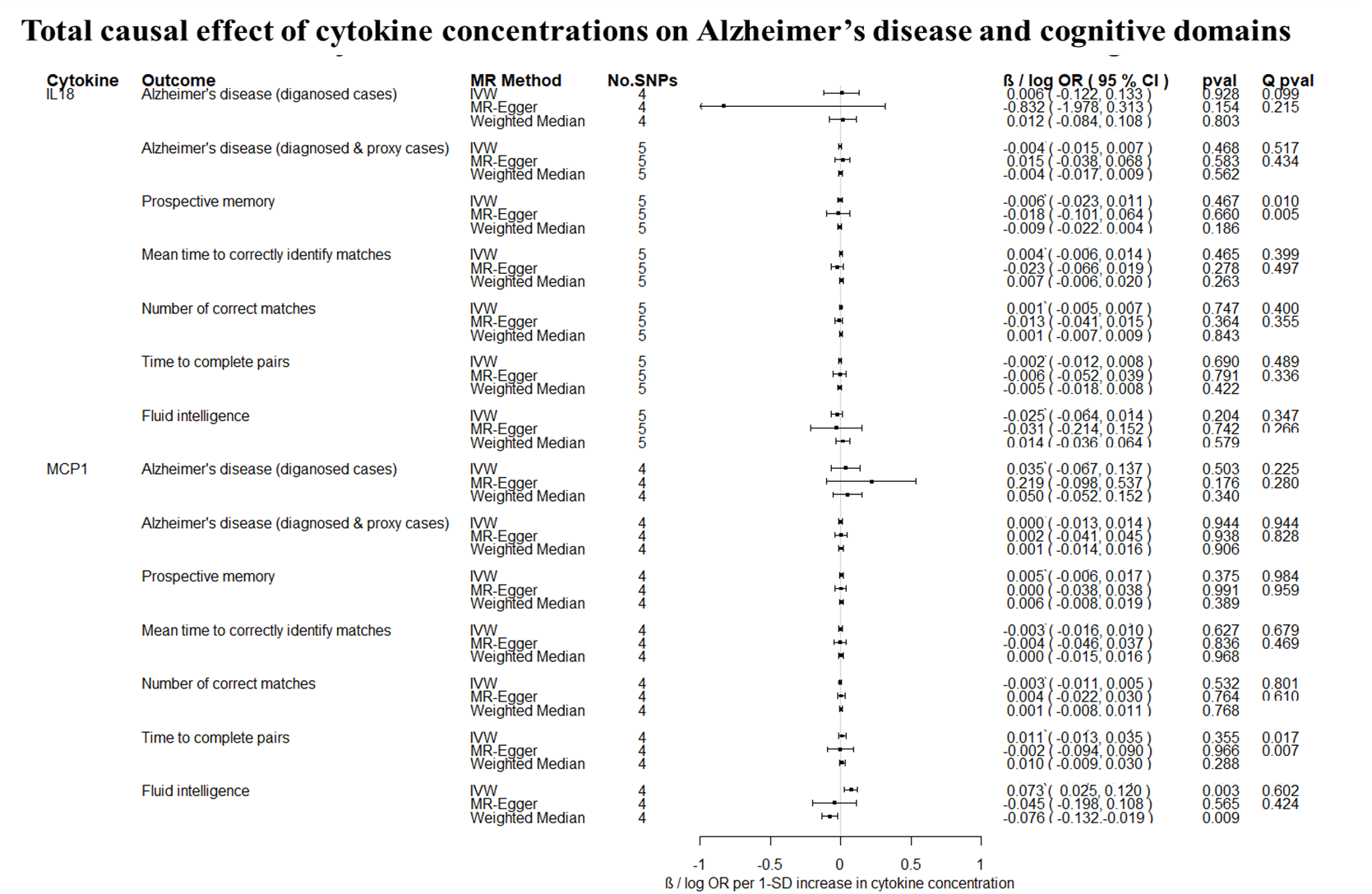 |
| --- |
| **Figure S1C. T**otal causal effects of circulating cytokine concentrations on the risk of Alzheimer’s disease and several cognitive outcomes, as estimated by Wald Ratio, IVW, MR-Egger and Weighted median estimators. |

| 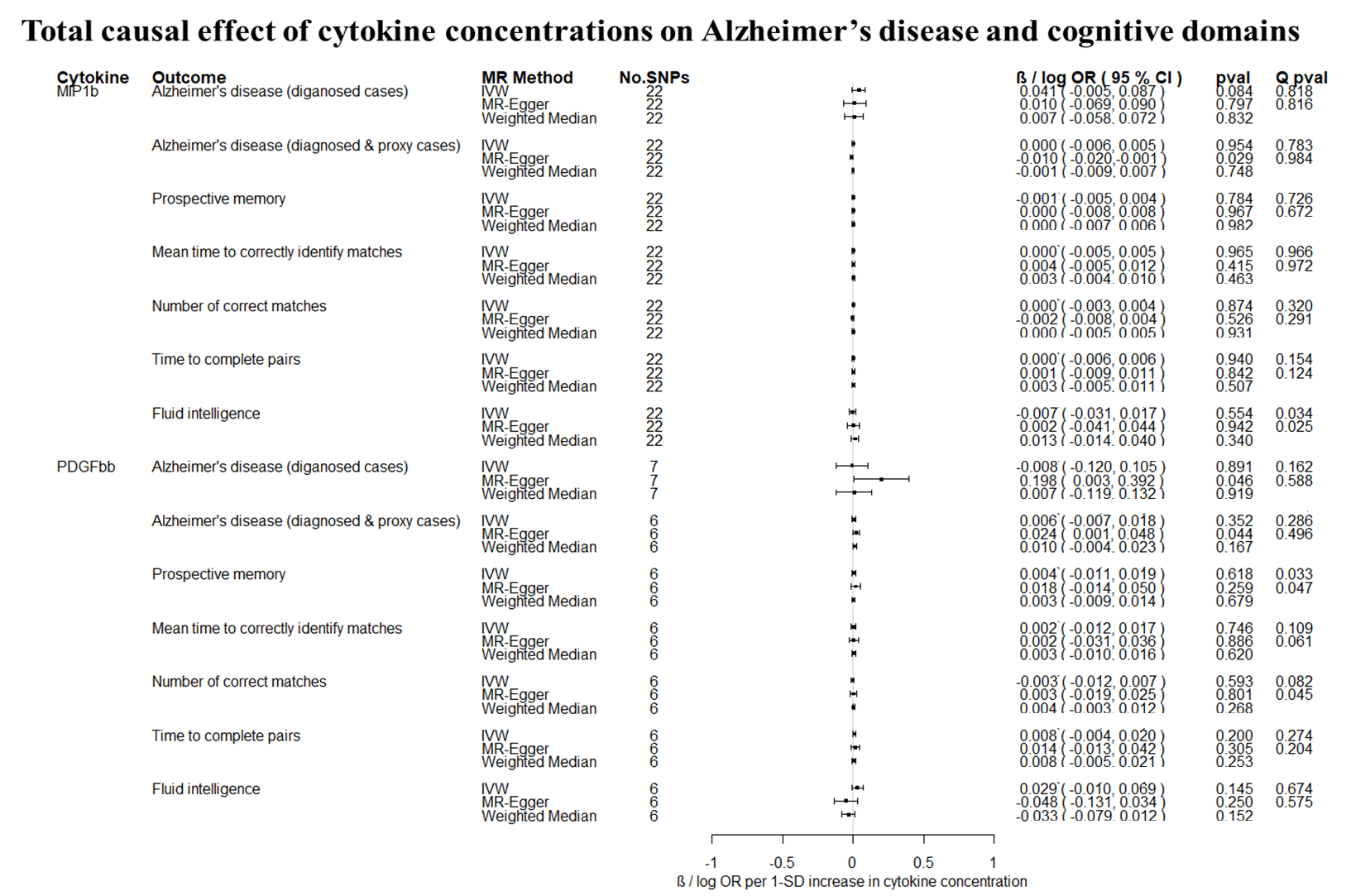 |
| --- |
| **Figure S1D.** Total causal effects of circulating cytokine concentrations on the risk of Alzheimer’s disease and several cognitive outcomes, as estimated by Wald Ratio, IVW, MR-Egger and Weighted median estimators |

| 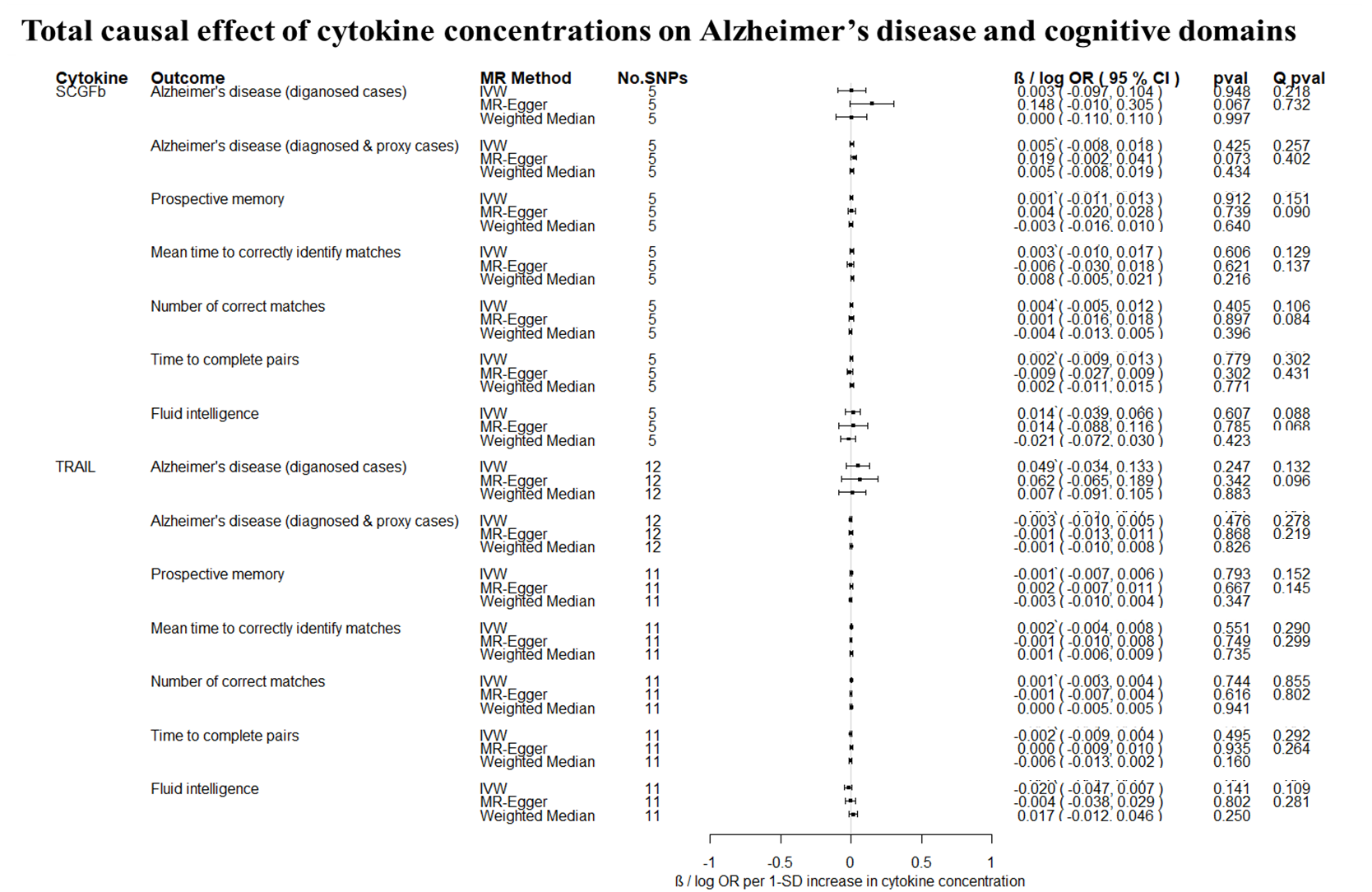 |
| --- |
| **Figure S1E. T**otal causal effects of circulating cytokine concentrations on the risk of Alzheimer’s disease and several cognitive outcomes, as estimated by Wald Ratio, IVW, MR-Egger and Weighted median estimators. |

| 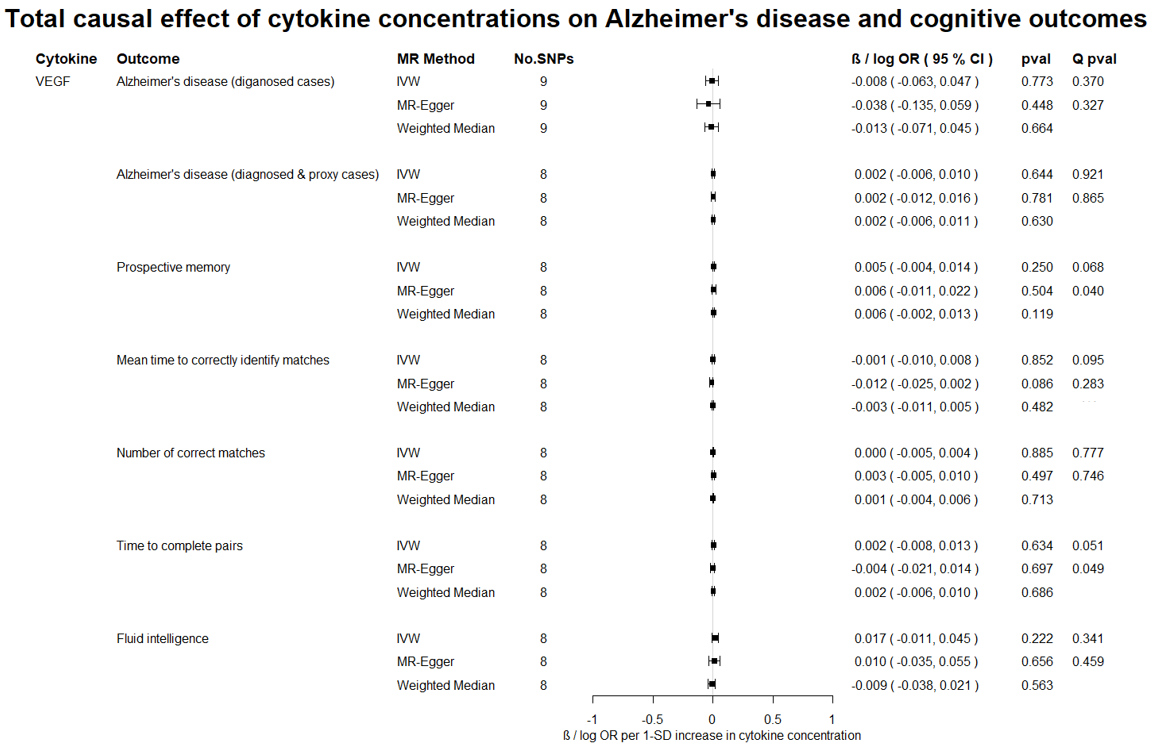 |
| --- |
| **Figure S1F. T**otal causal effects of circulating cytokine concentrations on the risk of Alzheimer’s disease and several cognitive outcomes, as estimated by Wald Ratio, IVW, MR-Egger and Weighted median estimators. |

| 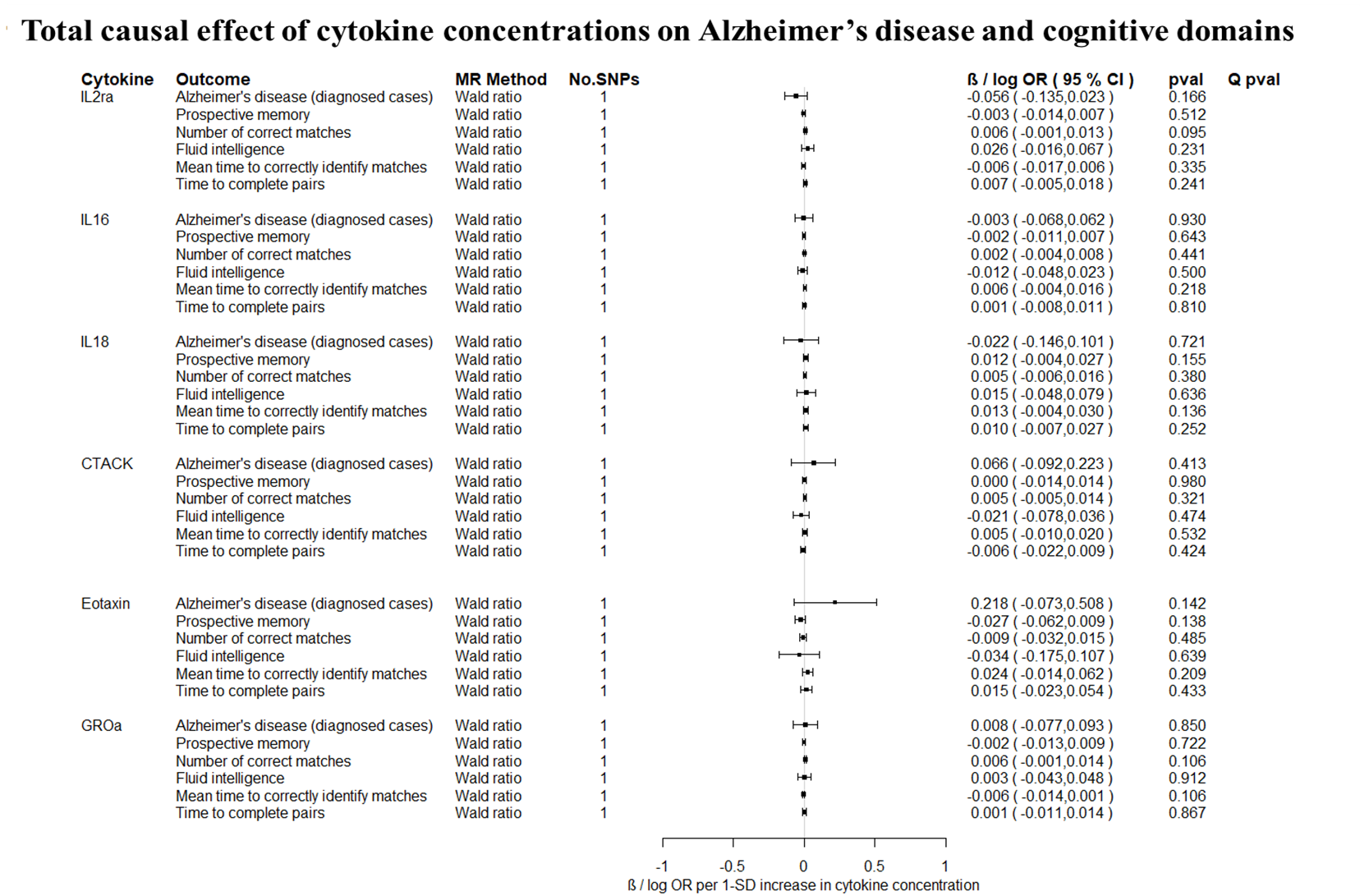 |
| --- |
| **Figure S2A.** Total causal effects of circulating cytokine concentrations on the risk of Alzheimer’s disease and several cognitive outcomes, as estimated by Wald Ratio and IVW estimators in the *cis* - Mendelian randomization analysis. |
| 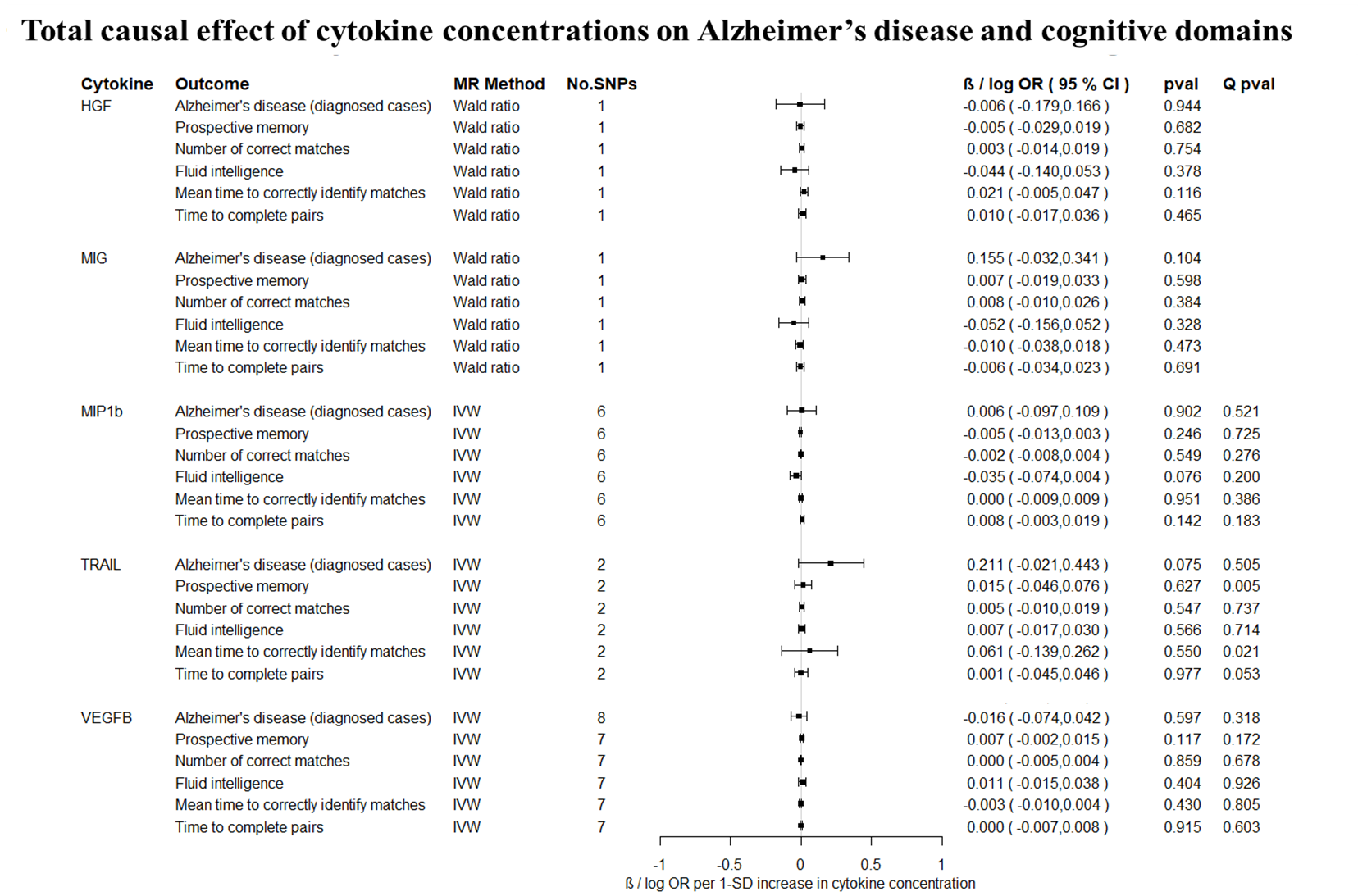 |
|  |
| **Figure S2B.** Total causal effects of circulating cytokine concentrations on the risk of Alzheimer’s disease and several cognitive outcomes, as estimated by Wald Ratio and IVW estimators in the *cis* - Mendelian randomization analysis. |

**REFERENCES**

1. Ahola-Olli AV, Wurtz P, Havulinna AS, et al. Genome-wide Association Study Identifies 27 Loci Influencing Concentrations of Circulating Cytokines and Growth Factors. Am J Hum Genet 2017;**100**(1):40-50 doi: 10.1016/j.ajhg.2016.11.007.
